# Data for proteomic profiling of Anthers from a photosensitive male sterile mutant and wild-type cotton (*Gossypium hirsutum* L.)

**DOI:** 10.1016/j.dib.2015.06.022

**Published:** 2015-07-09

**Authors:** Ji Liu, Chaoyou Pang, Hengling Wei, Meizhen Song, Yanyan Meng, Jianhui Ma, Shuli Fan, Shuxun Yu

**Affiliations:** a State Key Laboratory of Cotton Biology, Institute of Cotton Research, Chinese Academy of Agricultural Sciences, Anyang 455000, Henan Province, China; b Hubei Provincial Key Laboratory for Protection and Application of Special Plants in Wuling Area of China, College of Life Sciences, South Central University for Nationalities, Wuhan 430064, Hubei Province, China; c College of Life Sciences, Henan Normal University, Xinxiang 453007, Henan Province, China

**Keywords:** Photosensitive, Male sterility, Tapetum, Exine, Proteome, Pollen development

## Abstract

Cotton is an important economic crop, used mainly for the production of textile fiber. Using a space mutation breeding technique, a novel photosensitive genetic male sterile mutant CCRI9106 was isolated from the wild-type upland cotton cultivar CCRI040029. To study the male sterile mechanisms of CCRI9106, histological and iTRAQ-facilitated proteomic analyses of anthers were performed. This data article contains data related to the research article titled *iTRAQ-Facilitated Proteomic Profiling of Anthers From a Photosensitive Male Sterile Mutant and Wild-type Cotton (Gossypium hirsutum L.)*[1]. This research article describes the iTRAQ-facilitated proteomic analysis of the wild-type and a photosensitive male sterile mutant in cotton. The report indicated that exine formation defect is the key reason for male sterility in mutant plant. The information presented here represents the tables and figures that detail the processing of the raw data obtained from iTRAQ analysis.

**Table:** Specifications table.

Subject area	Biology
More specific subject area	Plant proteomics
Type of data	Table and figure
How data was acquired	Plant phenotype: DP72 light microscope (Olympus, Japan)
Scan electron microscopy: scanning electron microscopy S-530 (HITACHI, Japan)
Mass spectrometry: AB SCIEX Triple TOF 5600 System (AB SCIEX, USA)
Quantitative real-time PCR: ABI 7500 real-time PCR system (Applied Biosystems, USA)
Data format	Processed
Experimental factors	No pretreatment of samples was performed
Experimental features	Total anther protein was extracted from mutant and wild-type plants by triplicate using a TCA–acetone method. Three replicates iTRAQ-facilitated proteomic analysis were conducted for protein identification and quantification. Any protein changed with *a*≥1.5-fold difference and a *p*-Value≤0.05 in at least two replicates would thus be considered as a significant DEP in our data.
Data source location	Cotton anther samples were collected in Anyang, Henan Province, China. iTRAQ-facilitated proteomic analysis were conducted in Beijing Genomics Institute, Shenzhen, Guangdong Province, China.
Data accessibility	The mass spectrometry proteomics data have been deposited to the ProteomeXchange Consortium via the PRIDE partner repository with the dataset identifier PXD002209. The reviewer account: username, reviewer23539@ebi.ac.uk; password: 3ts0ERFU.

Value of the data

• An iTRAQ-based proteomic analysis in cotton anthers.
• Identification of 6,121 high-confidence proteins in cotton anther.
• There are 325 proteins show differential expression patterns between WT and MT.
• The data enrich the understanding of the molecular regulatory mechanisms of male sterility.

## 1 Experimental design

Using a space mutation breeding technique, a novel photosensitive genetic male sterile mutant CCRI9106 was isolated from the wild-type upland cotton cultivar CCRI040029. Histological and iTRAQ-facilitated proteomic analyses of anthers were performed to explore male sterility mechanisms of the mutant.

## 2 Materials and methods

### 2.1 Plant growth and anther collection

Two *G. hirsutum* L. genotypes, a PGMS mutant CCRI9106 and its WT line, CCRI040029, were used in this study. CCRI040029 was an elite upland variety bred in our lab, and the mutant line, CCRI9106, was created by space mutation in 2010 [2]. They were grown in an agronomic field in Anyang (Henan, China) from April to October (Fig. S1), and in Sanya (Hainan, China) from October to early April (Fig. S2). Thirty rows (8 m in length×0.8 m in width) were prepared for each genotype, and every 10 rows formed one replicate. To test the pollen fertility, anthers were stained with Alexander׳s solution. Additionally, anthers from both MT and WT at different development stages were collected for further analysis.

### 2.2 Scan electron microscopy

For SEM (Fig. S3), anthers were infiltrated with 2.5% (v/v) glutaraldehyde in phosphate buffer (0.1 M, pH 7.2), dehydrated in a graded series of ethanol (from 30% to 100%), treated in acetone for 15 min, and transferred to isoamyl acetate for 20 min. The samples were then dried with a CO_2_ critical-point drying system (HITACHI HCP-2, Japan). Subsequently, pollen grains were coated with gold:palladium and imaged using a scanning electron microscopy (HITACHI S-530, Japan).

### 2.3 Protein extraction and quantification

For protein extraction, a TCA–acetone (trichloroacetic acid) method [3] was selected, performed according to Pang et al. with minor modifications [4]. In brief, ~1.5 g of frozen anther was ground with 10% polyvinyl polypyrrolidone (w/w) in liquid nitrogen using a mortar and pestle. The resulting fine powder was mixed with 10% (w/v) TCA in cold acetone containing 0.07% (w/v) 2-mercaptoethanol for at least 2 h and subsequently centrifuged at 12,000 g for 1 h at 4 °C. The pellet was washed first with cold acetone containing 0.07% (w/v) 2-mercaptoethanol and then with 80% cold acetone and finally was suspended in lysis buffer (7 M urea, 2 M thiourea, 4% CHAPS, 20 mM dithiothreitol, 2% EDTA-free protease-inhibitor). The supernatant was centrifuged at 120,000 g for 90 min at 4 °C and used for further assays. Next, the purified proteins underwent a reductive alkylation reaction. The concentration of the protein solution was determined with the 2-D Quant Kit (GE Healthcare, USA) with bovine serum albumin as a standard. The supernatants were stored at –80 °C until required.

### 2.4 iTRAQ labeling

Three independent biological replicates were performed in our experiment (Fig. S4). Three internal standards (IS-1, IS-2, and IS-3) were prepared by mixing one biological replicate from the six tested samples. Then, proteins (100 μg) from each sample were digested by trypsin and labeled with 8-plex iTRAQ reagents (Applied Biosystems, USA) as follows: 113, IS; 114, IS; 115, WT-S1; 116, WT-S2; 117, WT-S3; 118, MT-S1; 119, MT-S2; 121, MT-S3. The labeled samples were pooled and resolved into 20 fractions using an Ultremex SCX column containing 5-μm particles (Phenomenex, USA). The eluted fractions were then desalted using a Strata X C18 column (Phenomenex, USA) and dried under vacuum. Each fraction was resuspended in certain volume of mobile phase A (2% ACN, 0.1% FA) and centrifuged at 20,000 g for 10 min. The final average peptide concentration in each fraction was about 0.25 μg/μL.

### 2.5 LC–MS/MS analysis

A splitless nanoACQuity (Waters, USA) system coupled with Triple TOF was used for analytical separation. The system uses microfluidic traps and nanofluidic columns packed with Symmetry C18 (5 μm, 180 μm×20 mm) for online trapping, desalting, and nanofluidic columns packed with BEH130 C18 (1.7 μm, 100 μm×100 mm) for analytical separations. Solvents were purchased from thermo fisher scientific and composed of water/acetonitrile/formicacid (A: 98/2/0.1%; B: 2/98/0.1%). A portion of 2.25 μg (9 μL) sample was loaded, and trapping and desalting were carried out at 2 μL/min for 15 min with 99% mobile phase A. At a flow rate of 300 nL/min, analytical separation was established by maintaining 5% B for 1 min. In the following 64 min, a linear gradient to 35% B occurred in 40 min. Following the peptide elution window, in 5 min the gradient was increased to 80% B and maintained for 5 min. Initial chromatographic conditions were restored in 2 min.

Data acquisition was performed with the AB SCIEX Triple TOF 5600 System (Concord, USA) fitted with a Nanospray III source (Concord, USA) and a pulled quartz tip as the emitter (New Objectives, Woburn, USA). Data was acquired using an ion spray voltage of 2.5 kV, curtain gas of 30 PSI, nebulizer gas of 15 PSI, and an interface heater temperature of 150 °C. The MS was operated with a RP greater than or equal to 30,000 FWHM for TOF MS scans. For IDA, survey scans were acquired in 250 ms and as many as 30 product ion scans were collected if exceeding a threshold of 120 counts per second (counts/s) and with a 2+ to 5+ charge-state. Total cycle time was fixed to 3.3 s. Q2 transmission window was 100 Da for 100%. Four time bins were summed for each scan at a pulser frequency value of 11 kHz through monitoring of the 40 GHz multichannel TDC detector with four-anode/channel detection. A sweeping collision energy setting of 35±5 eV coupled with iTRAQ adjust rolling collision energy was applied to all precursor ions for collision-induced dissociation. Dynamic exclusion was set for 1/2 of peak width (18 s), and then the precursor was refreshed off the exclusion list.

### 2.6 Database search and quantification

Protein identification and quantification were simultaneously performed using the Mascot 2.3.02 software (Matrix Science, Boston, USA). Searches were made against our cotton_AD_nr database, including 38,460 sequences from the *G. raimondii* genome [5] and 43,097 from the *G. arboretum* genome [6], the putative contributors of the D and A subgenomes, respectively, of the *G. hirsutum* L. genome (AADD). The search parameters were set as follows: trypsin was chosen as the enzyme with one missed cleavage allowed; the fixed modifications of carbamidomethylation were set as Cys, and variable modifications of oxidation as Met; peptide tolerance was set as 0.05 Da, and MS/MS tolerance was set as 0.1 Da. The peptide charge was set as Mr, and monoisotopic mass was chosen. An automatic decoy database search strategy was employed to estimate the false discovery rate (FDR). The FDR was calculated as the false positive matches divided by the total matches. In the final search results, the FDR was less than 1.5%. The iTRAQ 8-plex was chosen for quantification during the search. For protein identification, only peptides with significant scores (≥20) at the 99% confidence interval were used, and each confident protein included at least one unique peptide. For protein quantitation, “median” was chosen for the protein ratio type, only unique peptides were used to quantify proteins. The median intensities were set as normalization. We assigned the 6121 proteins detected from at least two replicates as finally identified proteins in this study (Table S1).

The mass spectrometry proteomics data have been deposited to the ProteomeXchange Consortium via the PRIDE partner repository [7] with the dataset identifier PXD002209.

We performed the analysis of biological replicates at each stage. The average CV of each stage ranges from 0.19–0.24, indicating high repeatability of our data (Table 1). Any protein changed with *a*≥1.5-fold difference and a *p*-Value≤0.05 in at least two replicates would thus be considered as a significant DEP in our data (Table S3).

### 2.7 Functional analyses

Functional category analysis (Table 2) was performed with Blast2GO software (http://www.geneontology.org) and Clusters of Orthologous Groups (COG) of Proteins System software (http://www.ncbi.nlm.nih.gov/COG/). To compare with Arabidopsis pollen proteome (3517 proteins from Arabidopsis pollen proteome analyses by Noir [8], Holmes-Davis [9] and Grobei [10]), all proteins in this study were blasted for the closest *Arabidopsis* homolog with *E*-value≤10^−10^ (Table S5). After a survey of the literatures, we updated a previously published list [11] of genes affected pollen development or pollen tube growth from 215 to 323 genes in *Arabidopsis* (Table S6).

### 2.8 RNA extraction and quantitative real-time PCR (qPCR)

To verify whether the differences in protein abundance were reflected at the transcriptional level, and to confirm the authenticity and accuracy of the proteomic analysis, 12 genes, one gene randomly selected from each cluster, were analyzed by qPCR at all three stages in WT and MT plants (Fig. S5). Total RNA from anther samples was extracted using the RN38-EASYspin Plus Plant RNA Kit (Aidlab, China) according to the manufacturer׳s protocol. Approximately 1 µg RNA was reverse transcribed to cDNA using SuperScriptIII (Invitrogen, USA) following its protocol. And qPCRs were carried out using SYBR Green PCR Master Mix (Roche Applied Science, Germany) on an ABI 7500 real-time PCR system (Applied Biosystems, USA) with three replicates. Data were processed using the 2^−ΔΔCt^ method, and the *GhUBQ7* (*GhUBQUTIN7*, DQ116441) was used as an endogenous reference gene and stage 1 was set as reference sample for data normalization. All the primer pairs used were shown in Table 3.

## Figures and Tables

**Table 1:** Analysis of the reproducibility between the three iTRAQ exprements of replicate samples.

**IS_113-VS-IS_114**				**Stage 1: WT_115-VS-MT_118**				**Stage 2: WT_116-VS-MT_119**				**Stage 3: WT_117-VS-MT_121**			
Cut-off at	Number	Total	Coverage (%)	Cut-off at	Number	Total	Coverage (%)	Cut-off at	Number	Total	Coverage (%)	Cut-off at	Number	Total	Coverage (%)
0.10	1126	2906	38.75	0.10	839	3109	26.99	0.10	982	2975	33.01	0.10	810	2702	29.98
0.20	2038	2906	70.13	0.20	1754	3109	56.42	0.20	1938	2975	65.14	0.20	1728	2702	63.95
0.30	2540	2906	87.41	0.30	2407	3109	77.42	0.30	2471	2975	83.06	0.30	2298	2702	85.05
0.40	2752	2906	94.70	0.40	2749	3109	88.42	0.40	2668	2975	89.68	0.40	2514	2702	93.04
0.50	2851	2906	98.11	0.50	2852	3109	91.73	0.50	2760	2975	92.77	0.50	2595	2702	96.04
0.60	2890	2906	99.45	0.60	2883	3109	92.73	0.60	2811	2975	94.49	0.60	2620	2702	96.97
0.70	2903	2906	99.90	0.70	2914	3109	93.73	0.70	2834	2975	95.26	0.70	2633	2702	97.45
0.80	2905	2906	99.97	0.80	2974	3109	95.66	0.80	2864	2975	96.27	0.80	2639	2702	97.67
0.90	2906	2906	100.00	0.90	2995	3109	96.33	0.90	2870	2975	96.47	0.90	2656	2702	98.30
>1.0	2906	2906	100.00	>1.0	3109	3109	100.00	>1.0	2975	2975	100.00	>1.0	2702	2702	100.00
CV (average)=0.17				CV (average)=0.24				CV (average)=0.21				CV (average)=0.19			

The table lists the cut-off points (variation), and the corresponding coverage (%) of quantified proteins.

a. “Cut off at” means the variation between the fold change and 1, and the fold change is calculated between two samples in the three experements.

b. “Number” means the number of proteins meet the cut off value.

c. “Total” means the total number of proteins quantified in at least two exprements.

d. “Coverage (%)” is calculated as the “Number” divided by the “Total”,and the higer coverage at a smaller cut off value means the better repeatability.

**Table 2:** Gene ontology (GO) enrichment analysis of DEPs from each stage.

GO category	GO term	Description	Cluster frequency	*P*-value	Proteins
Biological process	GO:0009651	Response to salt stress	5 of 10 in the list	0.0011	Cotton_D_gene_10020479,Cotton_D_gene_10026043,Cotton_A_02073,Cotton_D_gene_10040060,Cotton_A_15420
Biological process	GO:0010584	Pollen exine formation	2 of 10 in the list	0.0017	Cotton_D_gene_10020479,Cotton_A_15420
Biological process	GO:0016053	Organic acid biosynthetic process	5 of 10 in the list	0.0018	Cotton_D_gene_10020479,Cotton_A_02073,Cotton_D_gene_10040060,Cotton_A_15420,Cotton_A_15494
Biological p	GO:0009653	Anatomical structure morphogenesis	5 of 10 in the list	0.0021	Cotton_D_gene_10020479,Cotton_D_gene_10040060,Cotton_A_15420,Cotton_A_15494,Cotton_A_27442
Biological process	GO:0000097	Sulfur amino acid biosynthetic process	3 of 10 in the list	0.0035	Cotton_D_gene_10020479,Cotton_A_02073,Cotton_A_15420
Biological process	GO:0000096	Sulfur amino acid metabolic process	3 of 10 in the list	0.0046	Cotton_D_gene_10020479,Cotton_A_02073,Cotton_A_15420
Biological process	GO:0044283	Small molecule biosynthetic process	5 of 10 in the list	0.0049	Cotton_D_gene_10020479,Cotton_A_02073,Cotton_D_gene_10040060,Cotton_A_15420,Cotton_A_15494
Biological process	GO:0044711	Single-organism biosynthetic process	5 of 10 in the list	0.0065	Cotton_D_gene_10020479,Cotton_A_02073,Cotton_D_gene_10040060,Cotton_A_15420,Cotton_A_15494
Biological process	GO:0048869	Cellular developmental process	4 of 10 in the list	0.0074	Cotton_D_gene_10020479,Cotton_A_15420,Cotton_A_15494,Cotton_A_27442
Biological Process	GO:0045229	External encapsulating structure organization	3 of 10 in the list	0.0077	Cotton_D_gene_10020479,Cotton_A_15420,Cotton_A_15494
Biological process	GO:0044272	Sulfur compound biosynthetic process	3 of 10 in the list	0.0081	Cotton_D_gene_10020479,Cotton_A_02073,Cotton_A_15420
Biological process	GO:0009086	Methionine biosynthetic process	2 of 10 in the list	0.0087	Cotton_D_gene_10020479,Cotton_A_15420
Biological process	GO:0009751	Response to salicylic acid stimulus	2 of 10 in the list	0.0092	Cotton_A_02073,Cotton_D_gene_10040060
Biological Process	GO:0032989	Cellular component morphogenesis	3 of 10 in the list	0.0098	Cotton_D_gene_10020479,Cotton_A_15420,Cotton_A_27442
Biological process	GO:0006555	Methionine metabolic process	2 of 10 in the list	0.0100	Cotton_D_gene_10020479,Cotton_A_15420
Biological Process	GO:1901607	Alpha-amino acid biosynthetic process	3 of 10 in the list	0.0122	Cotton_D_gene_10020479,Cotton_A_02073,Cotton_A_15420
Biological process	GO:0006790	Sulfur compound metabolic process	3 of 10 in the list	0.0125	Cotton_D_gene_10020479,Cotton_A_02073,Cotton_A_15420
Biological Process	GO:0009067	Aspartate family amino acid biosynthetic process	2 of 10 in the list	0.0134	Cotton_D_gene_10020479,Cotton_A_15420
Biological process	GO:0010035	Response to inorganic substance	5 of 10 in the list	0.0143	Cotton_D_gene_10020479,Cotton_D_gene_10026043,Cotton_A_02073,Cotton_D_gene_10040060,Cotton_A_15420
Biological process	GO:0009309	Amine biosynthetic process	2 of 10 in the list	0.0162	Cotton_D_gene_10020479,Cotton_A_15420
Biological process	GO:0009409	Response to cold	3 of 10 in the list	0.0177	Cotton_D_gene_10026043,Cotton_A_02073,Cotton_D_gene_10040060
Biological process	GO:0009414	Response to water deprivation	2 of 10 in the list	0.0179	Cotton_D_gene_10026043,Cotton_D_gene_10040060
Biological process	GO:0009066	Aspartate family amino acid metabolic process	2 of 10 in the list	0.0189	Cotton_D_gene_10020479,Cotton_A_15420
Biological process	GO:0009415	Response to water stimulus	2 of 10 in the list	0.0189	Cotton_D_gene_10026043,Cotton_D_gene_10040060
Biological process	GO:0044767	Single-organism developmental process	5 of 10 in the list	0.0190	Cotton_D_gene_10020479,Cotton_D_gene_10040060,Cotton_A_15420,Cotton_A_15494,Cotton_A_27442
Biological process	GO:0019752	Carboxylic acid metabolic process	5 of 10 in the list	0.0227	Cotton_D_gene_10020479,Cotton_A_02073,Cotton_D_gene_10040060,Cotton_A_15420,Cotton_A_15494
Biological process	GO:0043436	Oxoacid metabolic process	5 of 10 in the list	0.0228	Cotton_D_gene_10020479,Cotton_A_02073,Cotton_D_gene_10040060,Cotton_A_15420,Cotton_A_15494
Biological process	GO:0006082	Organic acid metabolic process	5 of 10 in the list	0.0229	Cotton_D_gene_10020479,Cotton_A_02073,Cotton_D_gene_10040060,Cotton_A_15420,Cotton_A_15494
Biological process	GO:0009555	Pollen development	2 of 10 in the list	0.0237	Cotton_D_gene_10020479,Cotton_A_15420
Biological process	GO:0008652	Cellular amino acid biosynthetic process	3 of 10 in the list	0.0242	Cotton_D_gene_10020479,Cotton_A_02073,Cotton_A_15420
Biological process	GO:0048588	Developmental cell growth	2 of 10 in the list	0.0245	Cotton_A_15494,Cotton_A_27442
Biological process	GO:1901605	Alpha-amino acid metabolic process	3 of 10 in the list	0.0274	Cotton_D_gene_10020479,Cotton_A_02073,Cotton_A_15420
Biological process	GO:1901566	Organonitrogen compound biosynthetic process	4 of 10 in the list	0.0298	Cotton_D_gene_10020479,Cotton_A_02073,Cotton_D_gene_10040060,Cotton_A_15420
Biological process	GO:0009620	Response to fungus	2 of 10 in the list	0.0311	Cotton_A_02073,Cotton_D_gene_10040060
Biological process	GO:0016043	Cellular component organization	6 of 10 in the list	0.0317	Cotton_D_gene_10020479,Cotton_D_gene_10040060,Cotton_A_15420,Cotton_A_15494,Cotton_A_27442,Cotton_A_37611
Biological process	GO:0048646	Anatomical structure formation involved in morphogenesis	2 of 10 in the list	0.0356	Cotton_D_gene_10020479,Cotton_A_15420
Biological process	GO:0034641	Cellular nitrogen compound metabolic process	6 of 10 in the list	0.0356	Cotton_D_gene_10020479,Cotton_A_02073,Cotton_D_gene_10040060,Cotton_A_15420,Cotton_A_15494,Cotton_A_37611
Biological process	GO:0060560	Developmental growth involved in morphogenesis	2 of 10 in the list	0.0388	Cotton_A_15494,Cotton_A_27442
Biological process	GO:0048856	Anatomical structure development	5 of 10 in the list	0.0393	Cotton_D_gene_10020479,Cotton_D_gene_10040060,Cotton_A_15420,Cotton_A_15494,Cotton_A_27442
Biological process	GO:0044281	Small molecule metabolic process	6 of 10 in the list	0.0413	Cotton_D_gene_10020479,Cotton_A_14434,Cotton_A_02073,Cotton_D_gene_10040060,Cotton_A_15420,Cotton_A_15494
Biological process	GO:0071840	Cellular component organization or biogenesis	6 of 10 in the list	0.0438	Cotton_D_gene_10020479,Cotton_D_gene_10040060,Cotton_A_15420,Cotton_A_15494,Cotton_A_27442,Cotton_A_37611
Biological process	GO:0048589	Developmental growth	2 of 10 in the list	0.0467	Cotton_A_15494,Cotton_A_27442
Biological process	GO:0009308	Amine metabolic process	2 of 10 in the list	0.0478	Cotton_D_gene_10020479,Cotton_A_15420
Molecular function	GO:0080019	Fatty-acyl-CoA reductase (alcohol-forming) activity	2 of 10 in the list	0.0000	Cotton_D_gene_10020479,Cotton_A_15420
Molecular function	GO:0016491	Oxidoreductase activity	7 of 10 in the list	0.0003	Cotton_D_gene_10020479,Cotton_D_gene_10026043,Cotton_D_gene_10025048,Cotton_A_14434,Cotton_D_gene_10040060,Cotton_A_15420,Cotton_A_15494
Molecular function	GO:0003871	5-Methyltetrahydropteroyltriglutamate-homocysteine S-methyltransferase activity	2 of 10 in the list	0.0003	Cotton_D_gene_10020479,Cotton_A_15420
Molecular function	GO:0051213	Dioxygenase activity	2 of 10 in the list	0.0030	Cotton_D_gene_10025048,Cotton_D_gene_10040060
Molecular function	GO:0016620	Oxidoreductase activity, acting on the aldehyde or oxo group of donors, NAD or NADP as acceptor	2 of 10 in the list	0.0048	Cotton_D_gene_10020479,Cotton_A_15420
Molecular function	GO:0016614	Oxidoreductase activity, acting on CH-OH group of donors	3 of 10 in the list	0.0056	Cotton_D_gene_10026043,Cotton_A_14434,Cotton_A_15494
Molecular function	GO:0050662	Coenzyme binding	3 of 10 in the list	0.0080	Cotton_D_gene_10026043,Cotton_A_14434,Cotton_A_15494
Molecular function	GO:0016903	Oxidoreductase activity, acting on the aldehyde or oxo group of donors	2 of 10 in the list	0.0086	Cotton_D_gene_10020479,Cotton_A_15420
Molecular function	GO:0016705	Oxidoreductase activity, acting on paired donors, with incorporation or reduction of molecular oxygen	2 of 10 in the list	0.0129	Cotton_D_gene_10025048,Cotton_D_gene_10040060
Molecular function	GO:0008168	Methyltransferase activity	2 of 10 in the list	0.0174	Cotton_D_gene_10020479,Cotton_A_15420
Molecular function	GO:0016741	Transferase activity, transferring one-carbon groups	2 of 10 in the list	0.0188	Cotton_D_gene_10020479,Cotton_A_15420
Molecular function	GO:0016628	Oxidoreductase activity, acting on the CH-CH group of donors, NAD or NADP as acceptor	2 of 10 in the list	0.0208	Cotton_D_gene_10020479,Cotton_A_15420
Molecular function	GO:0048037	Cofactor binding	3 of 10 in the list	0.0246	Cotton_D_gene_10026043,Cotton_A_14434,Cotton_A_15494
Molecular function	GO:0016627	Oxidoreductase activity, acting on the CH-CH group of donors	2 of 10 in the list	0.0311	Cotton_D_gene_10020479,Cotton_A_15420
Molecular function	GO:0016616	Oxidoreductase activity, acting on the CH-OH group of donors, NAD or NADP as acceptor	2 of 10 in the list	0.0485	Cotton_D_gene_10026043,Cotton_A_15494
Cellular component	GO:0009941	Chloroplast envelope	4 of 11 in the list	0.0086	Cotton_D_gene_10020479,Cotton_A_02073,Cotton_D_gene_10040060,Cotton_A_15420
Cellular component	GO:0009526	Plastid envelope	4 of 11 in the list	0.0099	Cotton_D_gene_10020479,Cotton_A_02073,Cotton_D_gene_10040060,Cotton_A_15420
Cellular component	GO:0009536	Plastid	8 of 11 in the list	0.0115	Cotton_A_00728,Cotton_A_15420,Cotton_D_gene_10007359,Cotton_A_37611,Cotton_D_gene_10026043,Cotton_D_gene_10020479,Cotton_D_gene_10040060,Cotton_A_02073
Cellular component	GO:0044444	Cytoplasmic part	11 of 11 in the list	0.0289	Cotton_A_00728,Cotton_A_15420,Cotton_A_15494,Cotton_D_gene_10007359,Cotton_A_27442,Cotton_A_37611,Cotton_D_gene_10026043,Cotton_D_gene_10020479,Cotton_A_14434,Cotton_A_02073,Cotton_D_gene_10040060
Cellular component	GO:0005829	Cytosol	7 of 11 in the list	0.0289	Cotton_D_gene_10020479,Cotton_D_gene_10026043,Cotton_A_02073,Cotton_A_00728,Cotton_A_15420,Cotton_A_15494,Cotton_A_27442
Cellular component	GO:0009507	Chloroplast	7 of 11 in the list	0.0319	Cotton_D_gene_10020479,Cotton_D_gene_10026043,Cotton_A_02073,Cotton_D_gene_10040060,Cotton_A_00728,Cotton_A_15420,Cotton_D_gene_10007359
Cellular component	GO:0031967	Organelle envelope	4 of 11 in the list	0.0398	Cotton_D_gene_10020479,Cotton_A_02073,Cotton_D_gene_10040060,Cotton_A_15420
Cellular component	GO:0031975	Envelope	4 of 11 in the list	0.0398	Cotton_D_gene_10020479,Cotton_A_02073,Cotton_D_gene_10040060,Cotton_A_15420

DEPs are classified into three GO categories: biological process, molecular function and cellular component.

“Cluster Frequency” means number of DEPs in the list.

“*P*-value” means the reliability of each term, only terms with *P*-value<0.05 are shown.

“Proteins” are the DEPs annotated to the term.

**Table 3:** Primers sequences used for qPCR.

Primer name	Gene Name	Primer Sequences

Cotton_D_gene_10026043_F	*AKRC9*	GCCATATCGACTGCGCTCA
Cotton_D_gene_10026043_R	TGATAAACAGCTCCTCACGTT
Cotton_A_12079_F	*RPS23*	ACTCTGCCATCCGAAAGTGTG
Cotton_A_12079_R	CGCATGACCCTTTCGTCCA
Cotton_A_02073_F	*IPYR1*	CCCAAAGAGTCAAGTGCAAA
Cotton_A_02073_R	TTGCCCTTCTTAGTATCCTG
Cotton_A_23038_F	*AL2B4*	AGGGCTTCTATATTCAACCCACA
Cotton_A_23038_R	CCGAATAACCTCGTTGATGTCC
Cotton_A_20880_F	*ENPL*	TCCACGAGGAACACGCCTT
Cotton_A_20880_R	TCTCCTGCCATGTGTAAATCGG
Cotton_A_35622_F	*ACC1*	TTCTCTTTCTGTAAGGGGTC
Cotton_A_35622_R	TTTCCTTGCCAATAGACGTT
Cotton_A_01714_F	*CALM7*	GAATTCCTTAACCTGATGGCAAG
Cotton_A_01714_R	GTCAAACACCCTGAATGCCTC
Cotton_D_gene_10035730_F	*RBG8*	ATCCTCTGAATGTAAACCGAA
Cotton_D_gene_10035730_R	TTTTCTGCCTTGAATAATCAGC
Cotton_A_16087_F	*APX6*	TGCCATCCTATTCTGGTTCGT
Cotton_A_16087_R	TGCATGTTTCAGCTCGACT
Cotton_A_21984_F	*eiF2B5*	TTTACTTCAACAGCCAACCC
Cotton_A_21984_R	TCAATTTATCCGATCGAAGCTC
Cotton_D_gene_10039872_F	*PPI1*	TCACTTTTACCAGAGCCGTTC
Cotton_D_gene_10039872_R	GAGCCATAAACTTCTCGACCT
Cotton_D_gene_10027767_F	*Unknown*	AAAGCTCGTCTTGCCCGAT
Cotton_D_gene_10027767_R	TCCGAAAACCTGATTGCCCTT
Cotton_A_06160_F	*CO4C1*	AAAGGACTCTGCCTATCTCCA
Cotton_A_06160_R	TGTCTGGCTATTTTGAGCTTC
Cotton_D_gene_10008896_F	*CYP450*	CAGATAACAACTTCGCTCGG
Cotton_D_gene_10008896_R	CTTTCCAAGTAGAGCTTCGGA
Cotton_A_21314_F	*TKPR2*	CTCGCAACTCTAATTGATCCA
Cotton_A_21314_R	GCTTCTTGACATCGAAACGGTA
Cotton_A_15420_F	*MS2a*	CCAAGATCTATACCCGAGT
Cotton_A_15420_R	CATCCATATTTTCTAGCCCTT
Cotton_D_gene_10020479_F	*MS2b*	CTCCCTAGATTCGCCTTTGCTA
Cotton_D_gene_10020479_R	CACGGCCACTCTAAAGCTC
Cotton_D_gene_10018569_F	*QRT3*	AGCTCATTTCCTAGCCATGCC
Cotton_D_gene_10018569_R	AGCTTGATCCACCGTGACGA
Cotton_D_gene_10002752_F	*ABC26G*	TACAATCCGGCTCTTAAACGA
Cotton_D_gene_10002752_R	CAGGCTCATGTCACTCGGAA
Cotton_A_07399_F	*EA6*	AAATCGATCTCACCGGGAAC
Cotton_A_07399_R	TGCAAACATTTGACAATGCG
Cotton_D_gene_10029879_F	*SDR2A*	ACATTCATTGTGATGTAGCCAA
Cotton_D_gene_10029879_R	AAACAGTATGTCTAGTTTGCCTT
GhUB7-F1	*GhUB7*	TAGAGTCCGCTTCTACCTT
GhUB7-R1	ACGATTACGGAAAATCAAAGCC

## Footnotes

Appendix A Supplementary data associated with this article can be found in the online version at doi:10.1016/j.dib.2015.06.022.
