# Supplementary figures and images for: Data for proteomic profiling of Anthers from a photosensitive male sterile mutant and wild-type cotton (Gossypium hirsutum L.)
Source: Data Brief. 2015 Jul 9;4:500–9. doi: 10.1016/j.dib.2015.06.022 (PMC4773279; doi:10.1016/j.dib.2015.06.022)

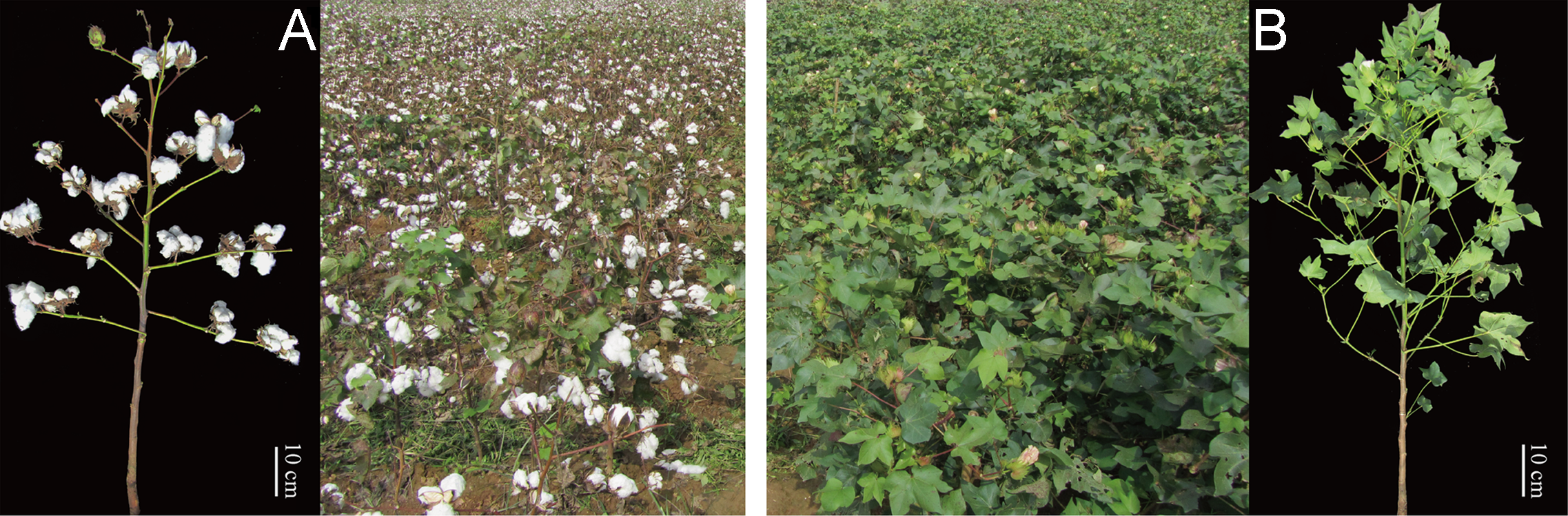

Supplement: Supplementary file 2 — Supplementary material [file mmc2.zip › mmc2/gr1.tif]

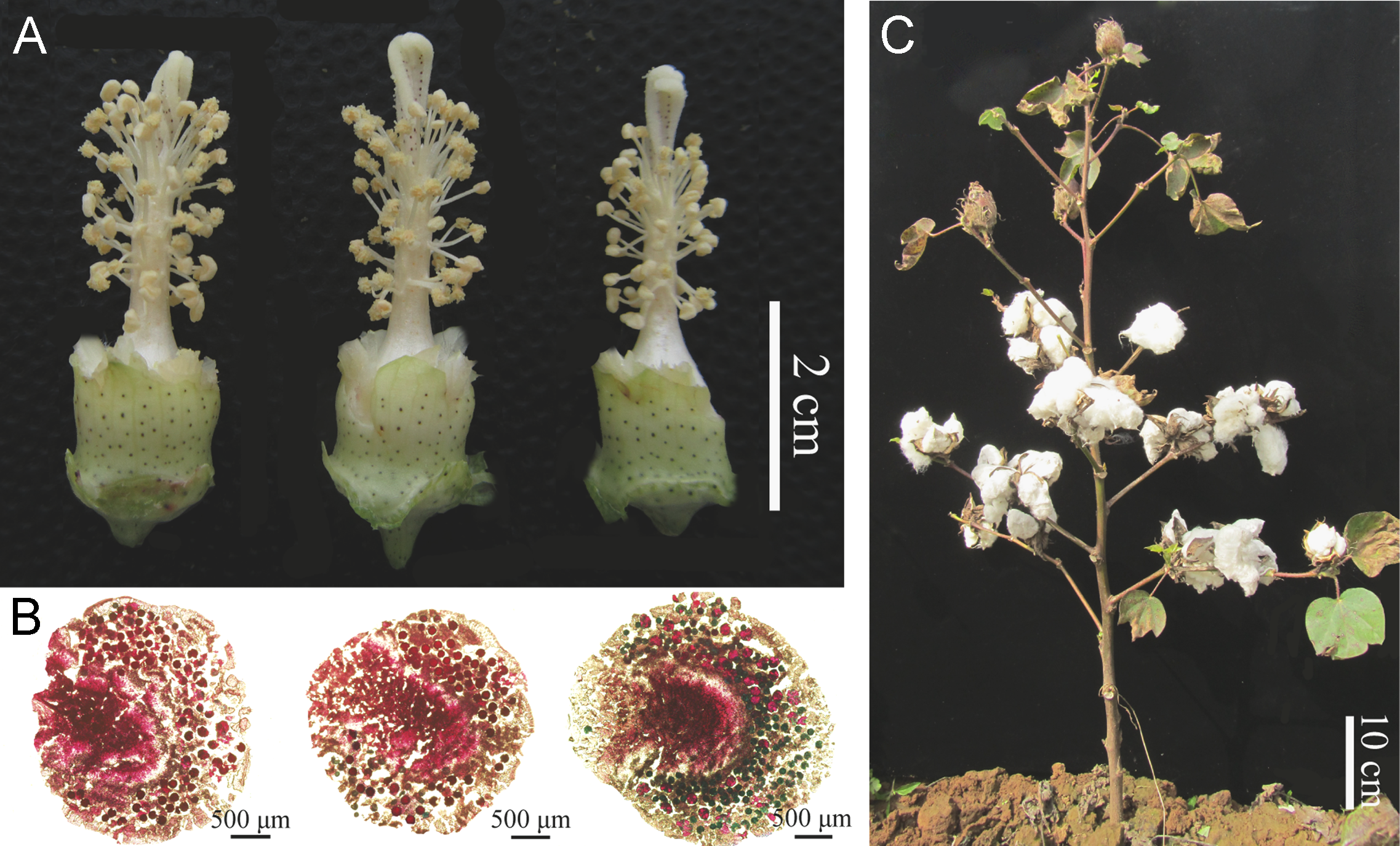

Supplement: Supplementary file 2 — Supplementary material [file mmc2.zip › mmc2/gr2.tif]

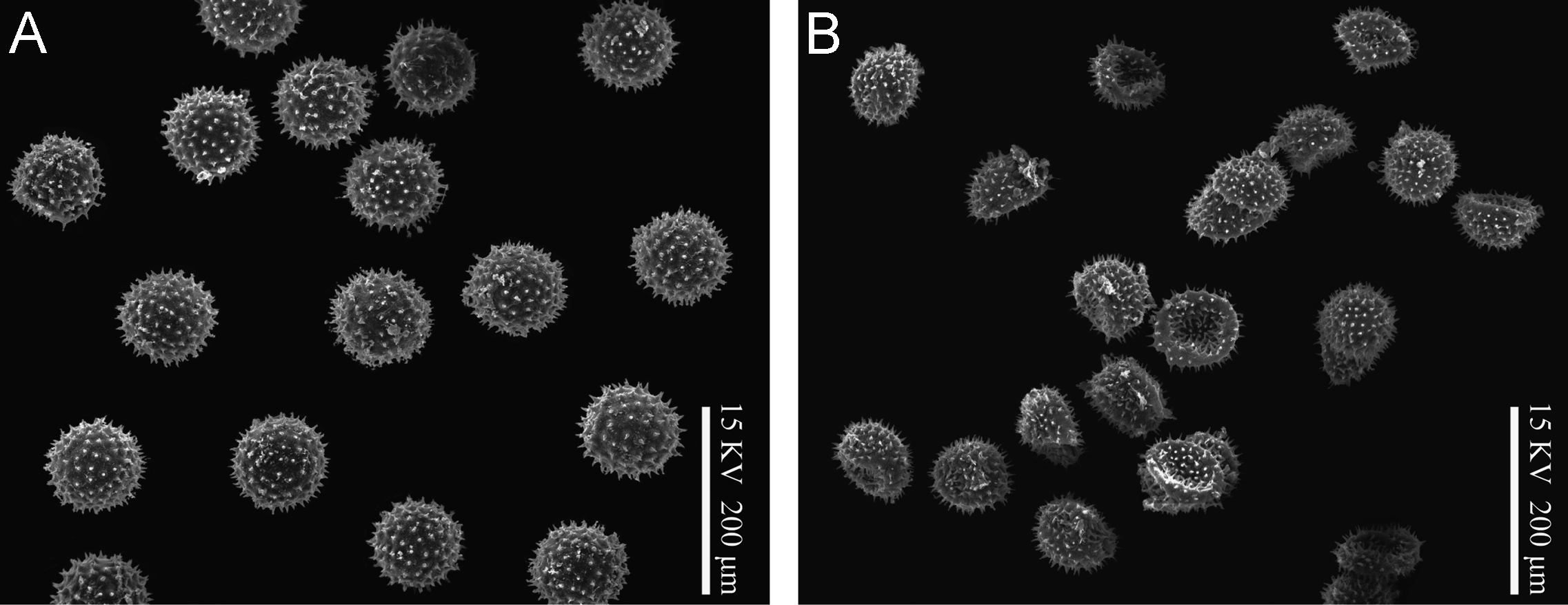

Supplement: Supplementary file 2 — Supplementary material [file mmc2.zip › mmc2/gr3.tif]

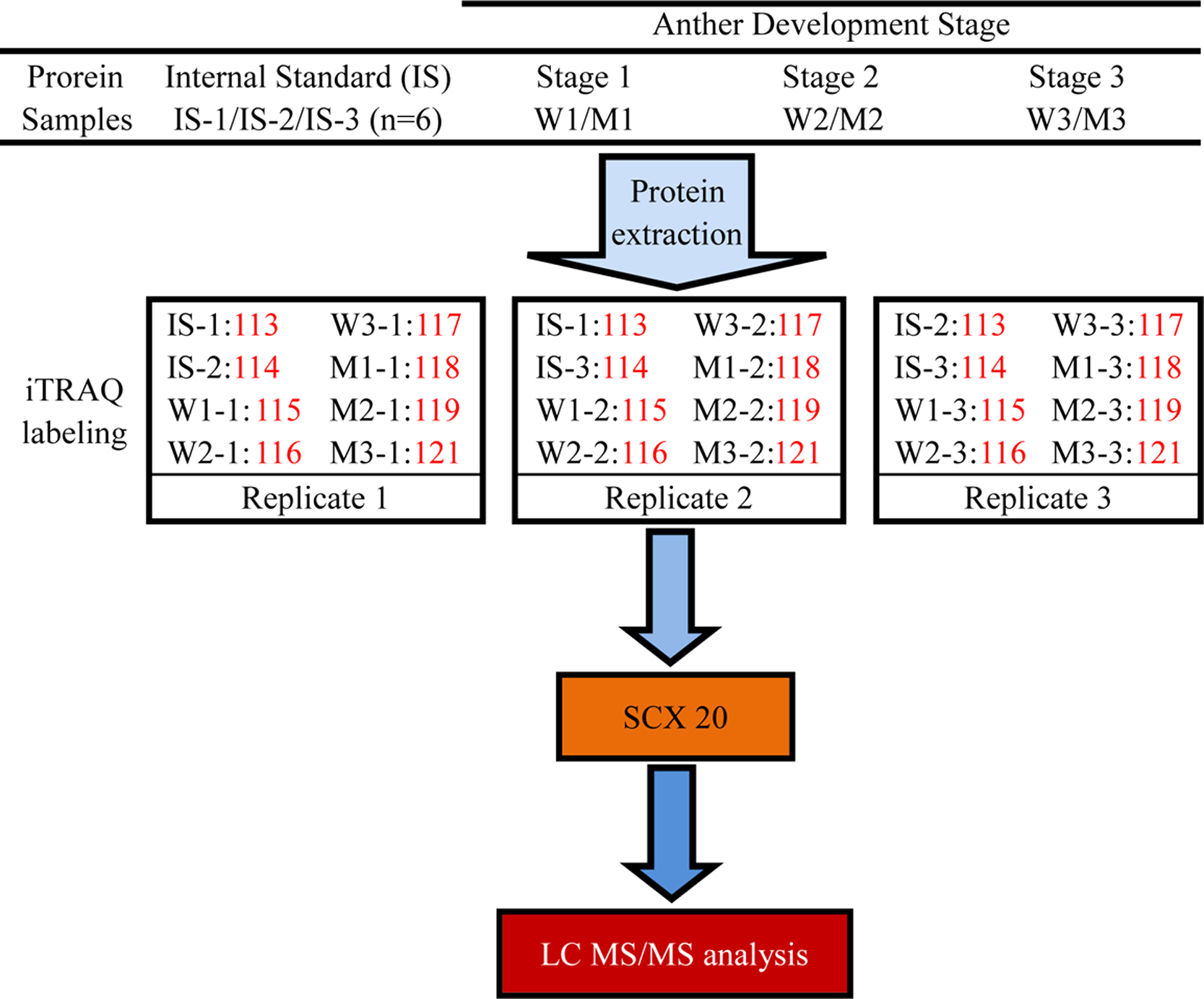

Supplement: Supplementary file 2 — Supplementary material [file mmc2.zip › mmc2/gr4.tif]

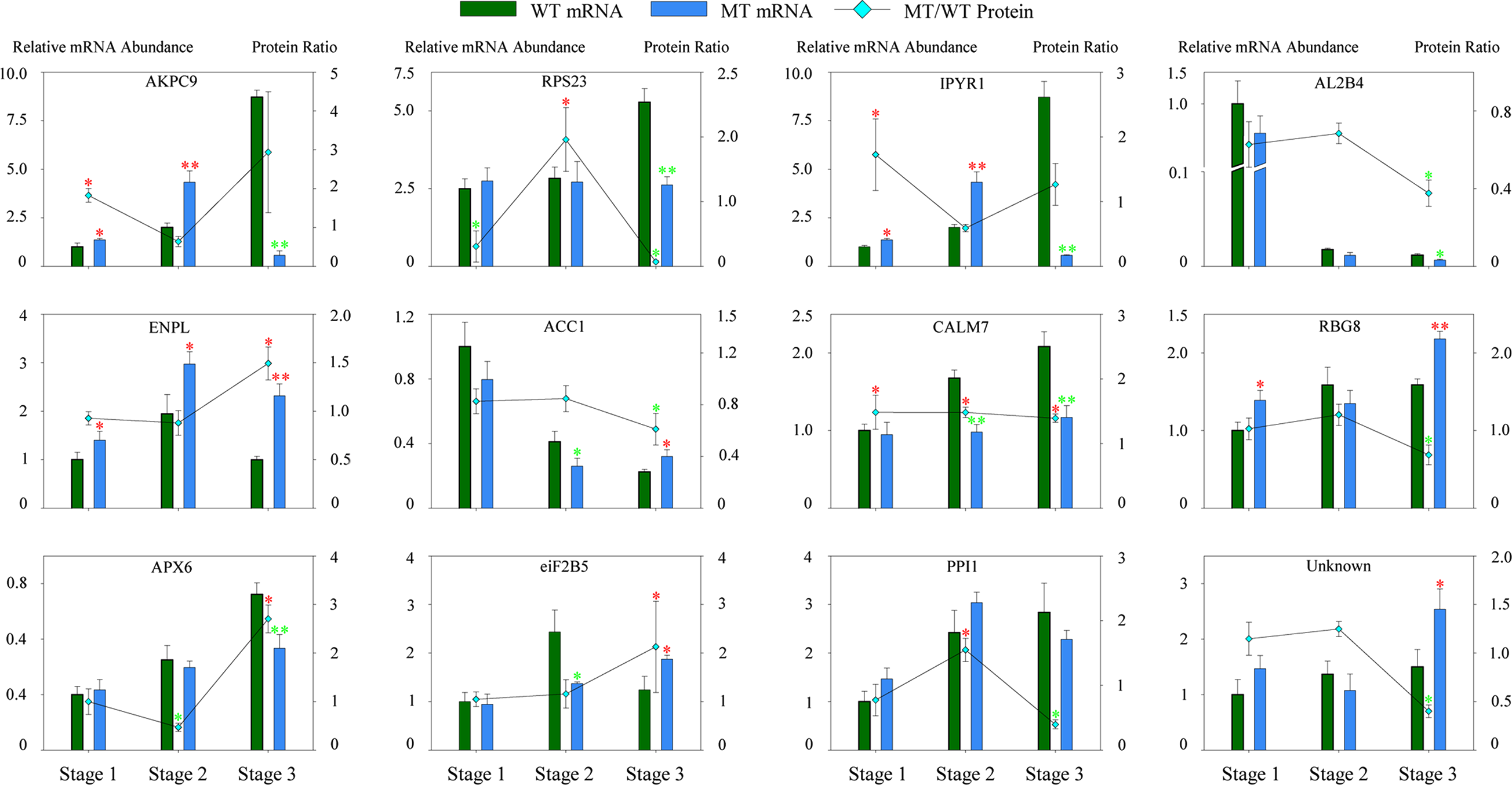

Supplement: Supplementary file 2 — Supplementary material [file mmc2.zip › mmc2/gr5.tif]
